# Intradermal Delivery of Catalase via Extracellular Vesicles for Targeted Photoaging Therapy

**DOI:** 10.34133/bmr.0329

**Published:** 2026-03-18

**Authors:** Pengchen Zhang, Bo Pan, Jiayi Chen, Hao Jiang, Xinhe Wang, Yiyou Chen, Wei Wang, Ming Li, Qingyu Zeng, Zhaogang Yang

**Affiliations:** ^1^ School of Life Sciences, Jilin University, Changchun 130012, China.; ^2^ Department of Dermatology, Shanghai Key Laboratory of Molecular Medical Mycology, Second Affiliated Hospital of Naval Medical University, Shanghai, China.; ^3^ Aesomed Bioscience Hong Kong Limited, Hong Kong, China.; ^4^ Department of Chemistry, University of Bergen, Bergen, Norway.; ^5^ Institute of Photomedicine, Shanghai Skin Disease Hospital, School of Medicine, Tongji University, Shanghai 200092, China.

## Abstract

The therapeutic potential of protein-based drugs is often limited by challenges in delivery, including instability, rapid degradation, and poor tissue targeting. Extracellular vesicles (EVs), as naturally derived nanocarriers, offer distinct advantages including biocompatibility, low immunogenicity, and efficient intercellular communication. Here, we engineered a 313 cell line to stably produce catalase (CAT)-loaded EVs (313EVs) that maintained vesicle integrity, exhibited high loading efficiency, and preserved enzymatic activity. Transcriptomic profiling revealed that genetic engineering subtly reshaped EV microRNA cargo, enriching 313EVs in pathways associated with EV uptake, mitochondrial membrane recovery, and DNA repair—supporting their multifaceted roles in mitigating photoaging. Functionally, 313EVs alleviated oxidative stress and restored antioxidant capacity in UVB-damaged fibroblasts. In vivo, intradermal administration resulted in sustained CAT activity, uniform dermal distribution, and marked improvements in wrinkle formation, collagen preservation, and skin elasticity. Notably, depletion of the skin microbiota did not alter therapeutic efficacy, indicating that the therapeutic benefits of 313EVs arise primarily from vesicle-intrinsic mechanisms rather than host–microbe interactions. Collectively, these findings establish 313EVs as a robust and versatile protein-delivery platform and highlight their therapeutic potential for combating oxidative stress-driven skin aging.

## Introduction

Photoaging is a degenerative skin process primarily driven by chronic ultraviolet (UV) radiation exposure, leading to the progressive accumulation of oxidative damage within dermal tissues [1]. This damage is largely mediated by an overproduction of reactive oxygen species (ROS), particularly hydrogen peroxide (H_2_O_2_), which promotes lipid peroxidation, DNA damage, and collagen degradation—key contributors to dermal atrophy, wrinkle formation, and loss of skin elasticity [2–6]. The pathophysiological consequences of ROS overproduction follow 3 primary pathways of tissue deterioration. First, lipid peroxidation, initiated by hydroxyl radicals derived from H_2_O_2_, targets polyunsaturated fatty acids in cellular membranes, generating cytotoxic aldehydes such as malondialdehyde that compromise epidermal barrier function [7,8]. Second, oxidative modifications of DNA bases, especially guanine, lead to the formation of mutagenic 8-oxo-2′-deoxyguanosine adducts, predisposing cells to genomic instability and impaired DNA repair in keratinocytes [8,9]. Third, the extracellular matrix (ECM) is disrupted via H_2_O_2_-induced activation of matrix metalloproteinases (MMPs), particularly MMP-1 and MMP-9, which degrade type I and III collagen fibrils essential for maintaining dermal structural integrity [10,11]. These biochemical alterations collectively manifest as coarse texture, deep wrinkles, and reduced skin elasticity—the hallmarks of photoaged skin [12].

Catalase (CAT), a tetrameric heme-containing enzyme predominantly localized in the peroxisomes of keratinocytes and dermal fibroblasts, serves as a frontline enzymatic defense against H_2_O_2_ overload [13]. Through its catalytic action, each CAT molecule can rapidly decompose millions of H_2_O_2_ molecules into water and molecular oxygen, thereby halting oxidative chain reactions before they propagate cellular damage [14]. Experimental studies have shown that CAT overexpression protects fibroblasts from UV-induced apoptosis, whereas its inhibition accelerates collagen degradation and promotes inflammatory responses [15–18]. However, paradoxically, CAT activity declines with age and cumulative photoexposure, as documented in several models of skin aging [19,20]. This enzymatic insufficiency leaves dermal tissues increasingly vulnerable to ROS-driven damage, exacerbating the progression of photoaging. CAT efficiently degrades UV-induced H_2_O_2_, thereby preventing ROS-mediated MMP up-regulation and subsequent collagen breakdown. Through this mechanism, CAT mitigates inflammation, restores redox homeostasis, and preserves the integrity of the dermal ECM. The therapeutic benefits of CAT in skin photoaging have been well documented. For example, a recent study developed a novel noninvasive transdermal delivery system based on chitosan-grafted sodium hexanoate (SCS) for efficient CAT delivery. The resulting SCS–CAT nanocomplex exhibited pronounced reparative and protective effects against UVB-induced skin photodamage.

Extracellular vesicles (EVs), including exosomes and microvesicles, are critical mediators in the transport of bioactive molecules, such as proteins and nucleic acids, across cells [21,22]. Given their inherent biocompatibility, ability to traverse physiological barriers, and low immunogenicity, EVs have emerged as attractive delivery vehicles for protein therapeutics. Unlike synthetic nanocarriers such as lipid nanoparticle (LNP), EVs are endogenously secreted by cells, reducing the risk of inflammatory responses [23]. Moreover, recent technological advancements have enabled the scalable and cost-effective production of EVs [24,25]. In the context of photoaging, EV-mediated delivery of antioxidant enzymes offers unique benefits. EVs can encapsulate and stabilize enzymatic proteins, support targeted uptake of cutaneous cells, and potentially enhance intracellular bioavailability of therapeutic antioxidants. Given that ROS accumulation is a central driver of photoaging, EV-mediated CAT delivery represents a rational and promising strategy to restore redox balance, suppress inflammation, and prevent collagen degradation.

In this study, we developed a kind of CAT-loaded EVs for targeted antioxidant therapy against photoaging. To generate these vesicles, we engineered the high-yield EV-producing 205 cell line (a HEK293 derivative) via lentiviral transfection with a human CAT expression construct, and subsequently isolated an optimized clone, designated 313, that retained robust EV secretion and produced CAT-enriched EVs (313EVs). We demonstrate that intradermal injection of 313EVs significantly attenuates oxidative stress, improves dermal antioxidant capacity, and mitigates structural and functional hallmarks of photoaging in UVB-exposed rats. These findings suggest that EV-mediated delivery of CAT may represent a promising and biocompatible strategy for the treatment and prevention of photoaging.

## Materials and Methods

### Cell culture

HFF-1 cells (American Type Culture Collection, SCRC-1041), HaCaT cells (The Cell Bank of the Chinese Academy of Sciences, SCSP-5091), and RAW 264.7 cells (Procell, CL-0190) were cultured in Dulbecco’s modified Eagle’s medium containing 10% fetal bovine serum and 1% penicillin–streptomycin at 37 °C with 5% CO_2_.

### Construction of 313EVs

The 205 cell line was obtained from Beijing Theraxyte Bioscience Co. Ltd. The detailed development and engineering procedures for the 205 cells are documented in the patent “*Methods for Manufacturing and Using Extracellular Vesicles*” (WO 2024/002311 A1).

The 313 cell line was generated from the 205 parental cells to enable high-efficiency loading of CAT into EVs. A recombinant expression vector was constructed using the backbone of Addgene plasmid #17448. This vector encodes wild-type human CAT and contains puromycin and penicillin resistance genes to facilitate antibiotic selection. The vector contains a puromycin resistance cassette for selection in mammalian cells and an ampicillin resistance cassette for plasmid propagation in *E. coli*. Lentiviral particles were produced by transfecting packaging cells with the recombinant transfer vector together with the packaging plasmids, and the resulting viral supernatant was used to transduce 205 cells. Following transduction, stable producer clones were selected with puromycin. Single-cell cloning and clone screening were subsequently performed to isolate cell lines with stable CAT expression and robust secretion of CAT-enriched EVs. EVs released from the engineered 313 cell line were collected and purified to obtain 313EVs.

### Confocal microscopy

Confocal microscopy was employed to explore the effect of loading CAT protein on endocytic efficiency by co-incubating labeled EVs with HFF-1 cells. After 4 h of treatment with PKH67-stained 313EVs and 205EVs, cells were washed and then fixed with 4% formaldehyde for 30 min. HFF-1 cell nuclei were stained with Hoechst33342, and cell fluorescence images were captured (FV3000, OLYMPUS, Japan).

### Flow cytometry analysis

HFF-1 cells were treated with PKH67-stained 313EVs and 205EVs for 4 h. After washing, the cells were fixed with 4% formaldehyde for 30 min. The fluorescence intensity of PKH26-labeled 313EVs and 205EVs uptake by HFF-1 cells was recorded using a flow cytometer (Cytoflex, Beckman, USA).

### 313EV pretreatment followed by H_2_O_2_-induced oxidative stress in HFF-1 cells in vitro

HFF-1 cells were preincubated with 1E10 particles/ml 313EVs for 24 h. After incubation, HFF-1 cells were treated with 150 μM H_2_O_2_ (Sigma-Aldrich, 88597-100ML-F) for 6 h. Then, the cells were replaced with EV-free medium and cultured for an additional 24 or 48 h.

### 313EV pretreatment followed by UVB-induced photoaging cell model in vitro

HFF-1 cells were divided into 3 groups: the NC group (without irradiation), the UVB Control group [treated with phosphate-buffered saline (PBS)], and the UVB 313EV-treatment group (treated with 1E10 particles/ml of 313EVs). In the UVB 313EV-treatment group, HFF-1 cells were preincubated with 313EVs for 24 h. After removing the EVs, both the UVB 313EV-treatment group and the UVB Control group were subjected to 150-mJ UVB irradiation. Post-irradiation, the cells were replaced with EV-free medium and cultured for an additional 24 or 48 h.

### Cell viability analysis

Cell viability of HFF-1 cells was assessed using the Cell Counting Kit-8 (CCK-8). HFF-1 cells were seeded in 96-well plates and cocultured with conditioned medium for 24 h. After irradiation with a dose of 150 mJ/cm^2^ of UVB, the CCK-8 reagent was added, and absorbance was measured at 595 nm.

### Wound scratch and transwell migration assays

HaCaT cells were plated and allowed to grow to a stable state. The 313EV-treatment group was first pretreated with 313EVs (1E10 particles/ml) for approximately 24 h (until the cells formed a relatively dense monolayer). Subsequently, both the Control group and the 313EV-treatment group were subjected to UVB irradiation at 100 mJ/cm^2^. After irradiation, a sterile pipette tip was used to create a scratch. The floating HaCaT cells were carefully washed away with PBS, and photographs of the scratch areas were taken under a microscope at 0 and 24 h. The area of the scratch was statistically analyzed to calculate the cell migration ability.

### γ-H2AX staining

After HFF-1 cells were plated and grew to a stable state, the 313EV-treatment group was pretreated with 313EVs (1E10 particles/ml) for 24 h. Subsequently, both the Control group and the 313EV-treatment group were subjected to UVB irradiation at 300 mJ/cm^2^, followed by continued culture for another 24 h. Cells were stained according to the instructions of the γ-H2AX kit. After staining, cells were observed, photographed, and statistically analyzed under a fluorescent microscope.

### SA-β-gal

After HFF-1 cells were plated and grew to a stable state, the 313EV group was pretreated with 313EV at a concentration of 1E10 particles/ml for 24 h. Subsequently, both the Control group and the 313EV group were subjected to UVB irradiation at 300 mJ/cm^2^, followed by continued culture for another 24 h. At the end of the culture, cells were stained according to the instructions of the SA-β-gal kit. After staining, cells were observed, photographed, and statistically analyzed under a microscope.

### Polarization of RAW 264.7 cells to M1 macrophages

The RAW 264.7 cell line, M0 macrophages, was polarized to M1 macrophages by treatment with 100 ng/ml lipopolysaccharide (LPS) and 20 ng/ml IFN-γ. The degree of M1 polarization was assessed by evaluating the levels of cytokines such as interleukin-6 (IL-6) and tumor necrosis factor-α (TNF-α). 313EVs were co-incubated with M1 macrophages to investigate their anti-inflammatory effects.

### Quantitative detection of intracellular H_2_O_2_ levels

The Peroxy Orange 1 (PO-1) reagent is a highly efficient and specific fluorescent probe used for the detection and quantification of intracellular H_2_O_2_ levels. HFF-1 cells were collected and prepared as cell suspensions, followed by staining with PO-1. The stained cells were then observed under an fluorescence microscope. The staining results were subjected to quantitative analysis and plotted.

### Enzyme-linked immunosorbent assay

Protein levels in cells and rat serum were detected using enzyme-linked immunosorbent assay (ELISA). The assay procedure was strictly followed according to the manufacturer’s instructions.

### RT-qPCR

HFF-1 cells and the dorsal skin tissues of Sprague–Dawley (SD) rats were collected, and total RNA was extracted using Trizol reagent. RNA was reverse-transcribed into cDNA following the protocol provided in the reverse transcription kit. For real-time quantitative polymerase chain reaction (RT-qPCR), the reaction mixture was prepared according to the qPCR kit protocol, containing the template, forward primer, reverse primer, qPCR SuperMix, and nuclease-free water. Fluorescent real-time amplification was then performed. Gene expression levels were quantified using the 2^−ΔΔCq^ method, with expression levels normalized to the housekeeping gene glyceraldehyde-3-phosphate dehydrogenase (GAPDH) [26].

### Animal models of photoaging

The female SD rats (6 weeks old, with an average weight of approximately 150 g) were purchased from Liaoning Changsheng Biotechnology Co. Ltd. The rats were acclimated for 1 week in the specific pathogen-free (SPF)-grade animal facility at the College of Life Sciences, Jilin University, under controlled conditions of 25 °C, with free access to water and food to adapt to the environment. The dorsal hair of SD rats was removed. All rats except the control group (*n* = 4) were exposed to UV irradiation to simulate photoaged skin conditions. The total doses of UVA and UVB were set at 14.07 and 11.66 J/cm^2^, respectively, with the UV lamp positioned 30 cm away. Rats received UV irradiation once daily for 7 weeks. Upon successful establishment of the photoaging model, UV-irradiated SD rats were randomly grouped and administered distinct drug treatments. The SD rats were randomly divided into the following groups: (a) NC group (without irradiation), (b) UV control group (saline group), (c) UV + 205EV group, (d) UV + 313EV low dose group (313EV-lo, 1E9 particles/dose), (e) UV + 313EV high dose group (313EV-hi, 1E10 particles/dose) [27,28], (f) UV + HA group, and (g) UV + RA group (*n* = 4 per group). The drug treatments were administered to the dorsal skin of rats on days 1, 5, 8, 15, and 22. EV groups and hyaluronic acid (HA) group were delivered via intradermal injection, and 0.5% RA was topically applied to the dorsal skin. During the experimental period, photographs were taken and the condition of the dorsal skin of each rat was documented regularly. At the end of the experiment, the rats were euthanized by cervical dislocation. Blood samples were collected from the orbital sinus of each rat, and the dorsal skin was excised and collected for subsequent analysis.

### Germ-reduced animal model for investigating microbiome–photoaging interactions

Germ-reduced rat models were established to evaluate whether the skin microbiota contributes to the therapeutic effects of 313EVs. Dorsal skin photoaging was induced in SD rats as described in the main experimental protocol. During the treatment period, 5% povidone–iodine, a broad-spectrum topical antiseptic, was applied to the dorsal skin twice daily to suppress resident microbial populations. Photographs were collected at predetermined time points to document changes in dorsal skin appearance throughout the experiment (*n* = 3 per group). Skin surface microbiota samples were obtained using sterile swabs and subjected to 16*S* ribosomal RNA (rRNA) gene sequencing to confirm microbial reduction and assess alterations in taxonomic composition.

### Wrinkle analysis

The photographs of the dorsal skin of the rats were imported into the image analysis software (Image Pro Plus), and wrinkle detection was performed using the software. The collected wrinkle data were subjected to statistical analysis to assess the construction of the photoaging model and the effects of treatment on wrinkle quantity.

### Hematoxylin and eosin staining

The SD rats were euthanized by cervical dislocation, and the dorsal skin tissues were excised using surgical scissors. Excess fat tissues were removed, and the skin samples were placed in centrifuge tubes containing 4% paraformaldehyde solution for fixation for 24 h. The fixed skin tissues were then removed from the tubes and embedded to prepare paraffin sections.

Hematoxylin and eosin (H&E) staining was performed, and the stained sections were examined under an optical microscope to observe the structure of each skin layer, as well as the presence of collagen and inflammatory cell infiltration within the skin.

### Masson’s trichrome staining

Paraffin sections were prepared and stained with Masson’s trichrome staining. The morphology and distribution of collagen fibers, as well as the collagen content, were examined under an optical microscope. The collagen content in the tissues was quantitatively assessed using ImageJ software.

### ROS analysis

SD rats were euthanized by cervical dislocation, and the dorsal skin tissues were excised using surgical scissors. Excess fat tissues were removed. The dihydroethidium (DHE) dye was dissolved in an appropriate solvent (e.g., dimethyl sulfoxide) to prepare a working solution of DHE at a specific concentration. Tissue sections were immersed in the DHE working solution, ensuring that the dye uniformly covered the sections. The sections were then incubated in the dark for a period of time to allow DHE to penetrate the cells and be oxidized by ROS. After incubation, the sections were washed with PBS or another appropriate buffer to remove unbound DHE dye.

The sections were observed under a fluorescence microscope with appropriate excitation and emission wavelengths set to detect the fluorescence signal generated by oxidized DHE. The intensity and distribution of the fluorescence signal within the cells were observed and recorded, with the fluorescence intensity being proportional to the level of ROS. The fluorescence signal was quantitatively analyzed using ImageJ software to assess the intracellular ROS levels.

### Western blotting

The total protein concentration in the skin was determined using a BCA protein assay kit. After adjusting to the desired concentration, protein samples were subjected to sodium dodecyl sulfate–polyacrylamide gel electrophoresis, followed by transfer of the protein bands to a polyvinylidene difluoride (PVDF) membrane. The PVDF membrane was incubated with the primary antibody overnight at 4 °C and then with the secondary antibody for 4 h at room temperature. Finally, the membrane was visualized using a gel imaging system (Bio-Rad, USA), with GAPDH serving as the reference protein.

### Statistical analysis

Quantitative data are presented as mean ± standard error of the mean (SEM). To analyze statistical differences between 2 groups, comparisons were made using a 2-sided Student’s *t* test. For comparisons involving more than 2 groups, one-way analysis of variance (ANOVA) followed by Tukey’s multiple comparisons test or Dunnett’s multiple comparisons test was used to assess statistical differences. *P* < 0.05 was considered statistically significant. Data were analyzed using GraphPad Prism 8.0.

## Results

### Characterization of 313EVs

**Fig. 1.**
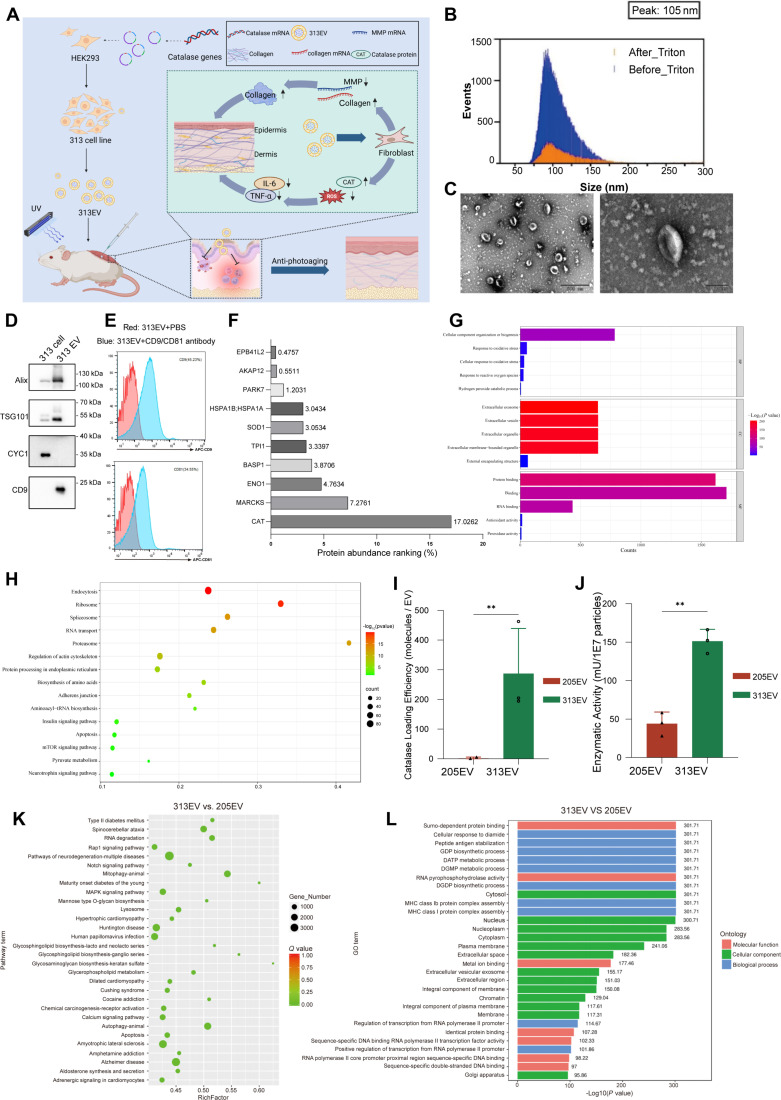
313EVs successfully encapsulate CAT protein. (A) Schematic representation of the engineered 313EV platform for targeted CAT delivery (created in BioRender). (B) Size distribution of 313EVs analyzed by nano-flow cytometry, revealing a peak at ~100 nm. (C) Transmission electron microscopy (TEM) image of 313EVs showing typical cup-shaped morphology. Scale bars, 500 nm (left) and 100 nm (right). (D) Western blot analysis confirming the presence of characteristic EV markers CD9, Alix, and TSG101. (E) Flow cytometry analysis verifying surface expression of EV markers CD9 and CD81. (F) Mass spectrometry analysis of 313EVs protein content confirming successful CAT loading. (G) Gene Ontology (GO) classification of identified proteins for biological process (blue), cellular component (red), and molecular function (yellow) demonstrates the antioxidant superiority of 313EVs. (H) Kyoto Encyclopedia of Genes and Genomes (KEGG) pathway reveals that 313EVs exert anti-photoaging effects via antioxidant pathways. (I) ELISA determination of CAT loading in 205EVs and 313EVs. (J) CAT enzyme activity in 205EVs versus 313EVs. (K) KEGG pathway analysis of miRNAs. (L) GO pathway analysis of miRNAs.

To generate CAT-loaded EVs, we genetically modified the 205 cell line by introducing the human CAT gene, thereby establishing the 313 cell line (Fig. 1A). Characterization of the 313EVs revealed a size distribution ranging from 60 to 150 nm, with a peak diameter of approximately 100 nm, as determined by nano-flow cytometry. Following treatment with Triton X-100, 89.15% of the particles were lysed, indicating that the majority were membrane-enclosed vesicles, confirming high purity of the EV preparation (Fig. 1B). Transmission electron microscopy (TEM) imaging showed the typical cup-shaped morphology of EVs, with diameters between 50 and 150 nm (Fig. 1C). Western blot analysis confirmed the presence of characteristic EV markers, including CD9, Alix, and TSG101 (Fig. 1D). In addition, flow cytometry analysis confirmed enrichment of surface markers CD9 and CD81, supporting the identity of the isolated vesicles as bona fide EVs (Fig. 1E). To confirm successful loading of CAT into 313EVs, proteins were extracted and analyzed by mass spectrometry, which identified a total of 1,445 proteins. Among them, CAT was highly ranked based on peptide score and intensity, confirming its effective incorporation into the 313EVs (Fig. 1F). Gene Ontology (GO) enrichment analysis revealed that, although both 205EVs and 313EVs were similarly enriched for pathways associated with basic cellular component organization, only 313EVs showed enrichment for extracellular exosome/vesicle components and external encapsulating structure pathways. This unique profile supports more effective delivery of CAT to regions of active peroxidase activity. Moreover, 313EVs were specifically enriched for antioxidant activity and hydrogen peroxide catabolic processes, enabling direct neutralization of UVB-induced ROS—functional attributes that were absent in 205EVs. In parallel, 313EVs enhanced pathways related to cellular responses to oxidative stress, supported by RNA- and protein-binding functions that stabilize CAT mRNA and preserve the structural integrity of the enzyme (Fig. 1G). Kyoto Encyclopedia of Genes and Genomes (KEGG) pathway analysis further indicated activation of antioxidant-associated pathways, including apoptosis, mechanistic target of rapamycin (mTOR) signaling, pyruvate metabolism, and neurotrophin signaling (Fig. 1H). Among all differentially expressed proteins between 205EVs and 313EVs, CAT exhibited the largest change, with a log_2_ fold change of 18.5 (Table S1). ELISA and CAT activity assays confirmed markedly elevated CAT expression and enzymatic activity in 313 cells compared with 205 cells, consistent with high CAT abundance and functional activity in 313EVs (Fig. 1I and J).

To evaluate whether genetic engineering altered vesicular RNA content, we performed RNA sequencing to profile microRNAs (miRNAs) in 205EVs and 313EVs. KEGG analysis revealed that 313EV miRNAs were enriched in pathways related to EV uptake, such as ECM–receptor interaction, endocytosis, adherens junctions, tight junctions, and focal adhesion, suggesting efficient internalization by keratinocytes and fibroblasts. These pathways support delivery of membrane lipids and glycosphingolipid precursors to damaged tissue, promoting barrier glycosylation repair and collagen fiber remodeling. Additionally, enrichment in membrane phospholipid metabolism pathways indicates that 313EVs may supply substrates essential for mitochondrial membrane restoration and recovery of UVB-depleted adenosine triphosphate (ATP) synthesis (Fig. 1K). GO enrichment analysis demonstrated that the miRNA-associated functional modules align closely with biological processes involved in photoaging repair. Nuclear-related terms, including SUMO-dependent protein binding, chromatin remodeling, and RNA polymerase II-mediated transcriptional regulation, suggest that EV-delivered miRNAs primarily fine-tune DNA repair rather than induce proto-oncogenic transcription. Furthermore, the low abundance of components associated with major histocompatibility complex (MHC) class I/II antigen assembly and peptide loading indicates minimal immunogenic risk and absence of pathways that drive chronic inflammation in photoaged skin (Fig. 1L).

Collectively, these results demonstrate that genetic modification of 205 cells not only enables efficient CAT loading but also subtly remodels the miRNA cargo of their secreted vesicles. The resulting 313EVs are enriched in functional modules that mitigate oxidative damage, enhance DNA repair, and promote barrier reconstruction—contributing synergistically to their therapeutic efficacy in photoaging.

### Effects of 313EVs on photoaged cells in vitro

**Fig. 2.**
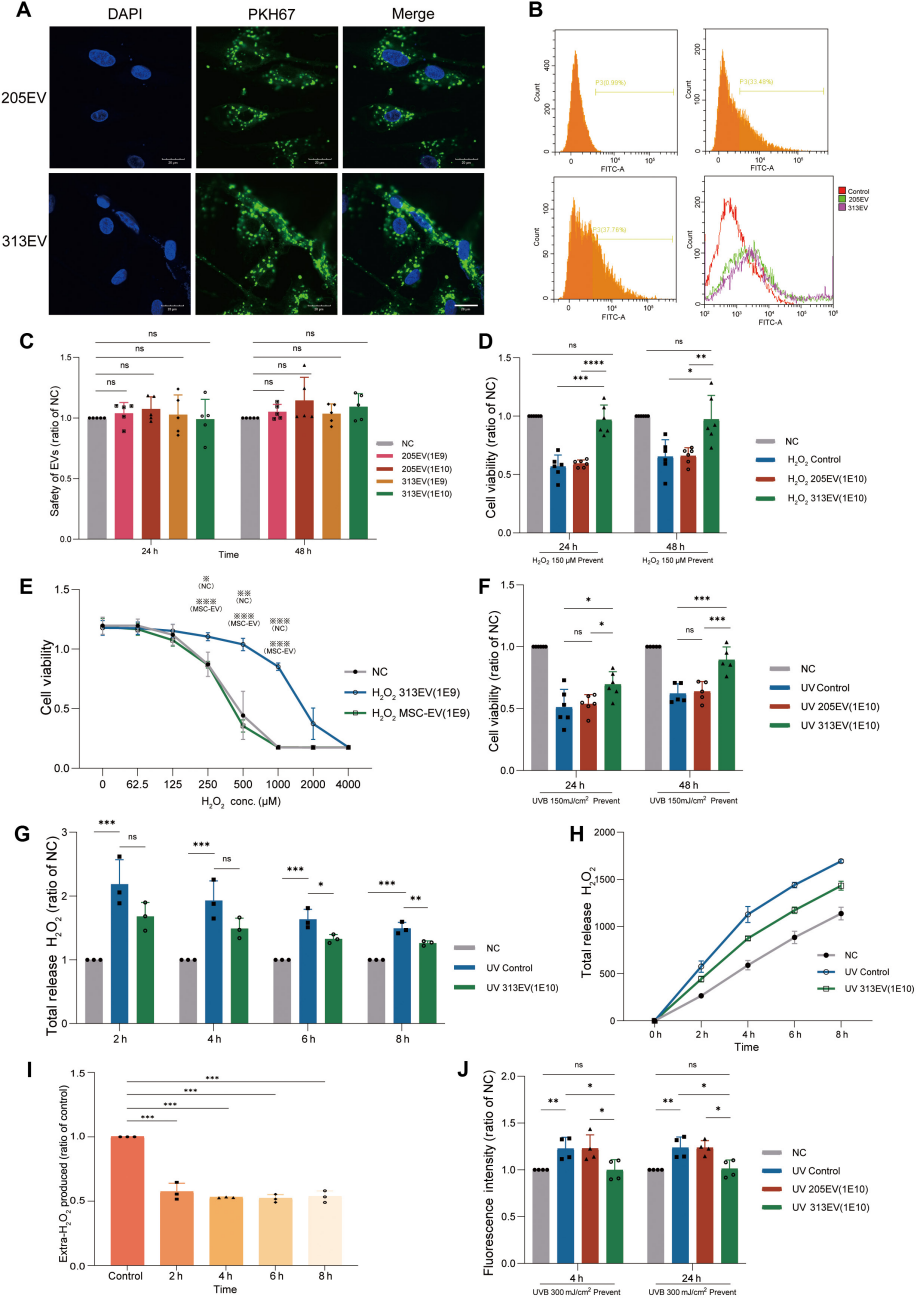
313EVs mitigate photoaging-induced damage in HFF-1 cells in vitro. (A) Confocal fluorescence microscopy demonstrated colocalization of PKH67-labeled EVs with HFF-1 cells. Scale bar, 20 μm. (B) Fluorescence intensity of PKH67-labeled EVs taken up by HFF-1 cells was measured by flow cytometry. (C) CCK-8 quantitatively assessed cytotoxicity of the EVs and the proliferation of HFF-1 cells (*n* = 5). (D) Antioxidant capacity of 313EVs assessed in HFF-1 cells subjected to high-dose H_2_O_2_-induced oxidative stress (*n* = 3). (E) Comparison of cell viability in H_2_O_2_-treated HFF-1 cells following treatment with 313EVs and huMSC-EVs. (F) Cell viability of HFF-1 cells treated with 313EVs in UVB-induced damage model (*n* = 3). (G and H) Quantification of total H_2_O_2_ released from HFF-1 cells after 313EV treatment in the UVB model (*n* = 3). (I) Measurement of extracellular H_2_O_2_ levels in UVB-treated HFF-1 cells following 313EV treatment (*n* = 3). (J) Fluorescence intensity of PO-1-stained HFF-1 cells detected by microplate reader (*n* = 3). *P < 0.05, **P < 0.01, ***P < 0.001.

To analyze the protective effect of 313EVs against UV-induced oxidative damage, we first evaluated their cellular uptake in HFF-1. PKH67-labeled 313EVs were effectively internalized by HFF-1 cells, with no significant difference in uptake efficiency compared to 205EVs, indicating that CAT enrichment did not interfere with vesicle internalization (Fig. 2A). Furthermore, flow cytometry analysis corroborated this result, showing comparable uptake levels between 313EVs and 205EVs (Fig. 2B). To evaluate the cytotoxicity of 313EVs in vitro, HFF-1 fibroblasts were treated with various concentrations of different exosome types and analyzed using a CCK-8 assay. The results showed no significant difference in cell viability between the 205EV- and 313EV-treated, and negative control (NC) groups, confirming the biocompatibility and low cytotoxicity of both EV formulations, even at high doses (Fig. 2C). To assess the antioxidant capacity of 313EVs, HFF-1 cells were treated with 313EVs prior to exposure to H_2_O_2_, a common inducer of oxidative stress. After 24 and 48 h, H_2_O_2_ treatment significantly reduced cell viability to 56.78% and 65.36%, respectively, compared to untreated controls. In contrast, cells pretreated with 313EVs (1E10 particles/ml) showed a marked improvement in viability—increasing it by 70.5% at 24 h and 48.8% at 48 h compared to the control group—demonstrating substantial antioxidant protection (Fig. 2D). To further verify this protective role, 313EVs were compared with high-purity EVs derived from human mesenchymal stem cells (huMSCs) under increasing concentrations of H_2_O_2_. The 313EV-treated group consistently exhibited higher cell viability than the huMSC-EV group, confirming superior cytoprotective efficacy (Fig. 2E). The anti-photoaging effect of 313EVs was next examined under UV-induced stress. HFF-1 cells were exposed to 150 mJ/cm^2^ UVB irradiation, resulting in significant viability loss to 50% at 24 h and 38% at 48 h. Pretreatment with 313EVs substantially mitigated this damage, with cell viability increasing to 20% and 44% at 24 and 48 h, respectively (Fig. 2F). The impact of 313EVs on oxidative stress was further evaluated by quantifying extracellular H_2_O_2_ levels post-UVB irradiation. Compared to the UVB-only group, 313EV pretreatment significantly reduced H_2_O_2_ release by 18.7% at 6 h and 15.5% at 8 h (Fig. 2G and H). A broader time-course analysis confirmed a consistent suppression of extracellular H_2_O_2_ levels at 2 h (42.8%), 4 h (47.2%), 6 h (48.0%), and 8 h (46.5%) post-UVB exposure (Fig. 2I). Microplate reader was used to detect the fluorescence signal of HFF-1 cells stained with PO-1 after UVB irradiation. Remarkably, both results indicate that 313EV pretreatment effectively mitigated this oxidative surge, maintaining intracellular H_2_O_2_ concentrations within near-physiological ranges (Fig. 2J).

### Effects of 313EVs on gene expression and protein secretion in vitro

**Fig. 3.**
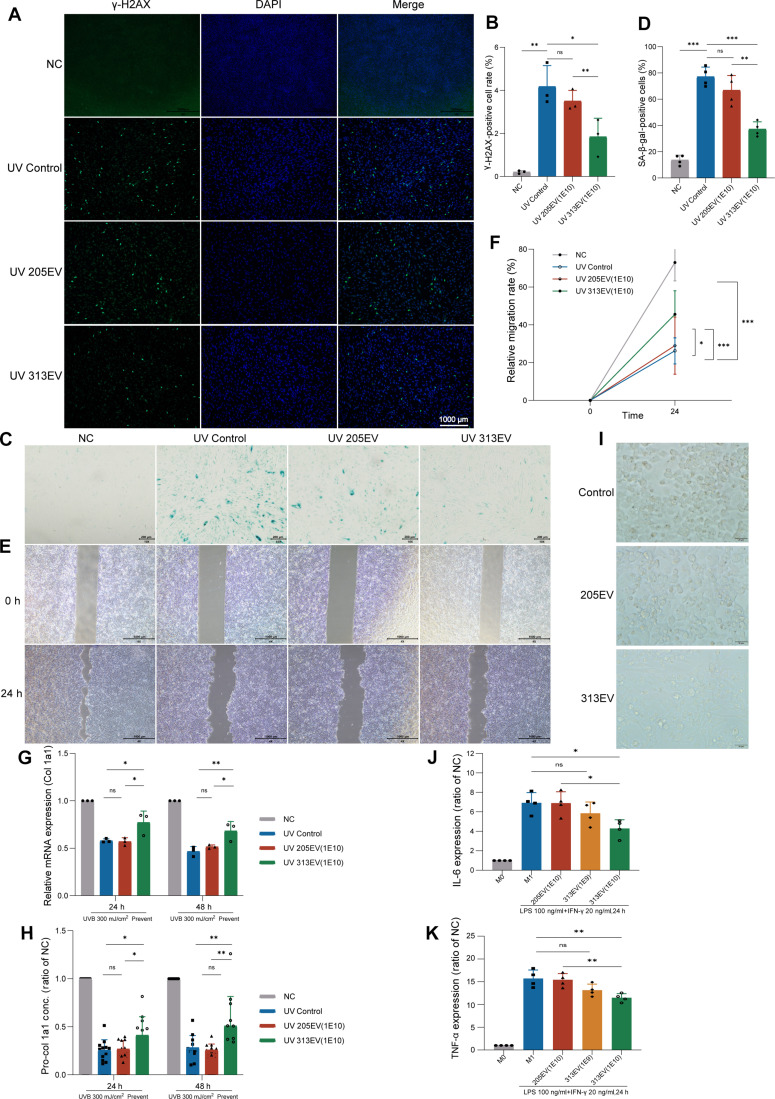
313EVs alleviate photoaging damage and inflammation in HFF-1 cells in vitro. (A) γ-H2AX staining shows the degree of cellular DNA damage. Scale bar, 1,000 μm. (B) Quantification of the fluorescence intensity of γ-H2AX staining (*n* = 3). (C) SA-β-gal staining shows the degree of cellular senescence. Scale bar, 200 μm. (D) Quantification of the degree of cellular senescence from SA-β-gal staining (*n* = 3). (E) HaCaT scratch assay shows the degree of cell migration, with photographs taken under a microscope. Scale bar, 1,000 μm. (F) Statistical analysis of the scratch area to calculate the cell migration ability (*n* = 3). (G) Quantitation of *COL1A1* mRNA expression in HFF-1 cells by RT-qPCR after UVB exposure and 313EV treatment (*n* = 3). (H) Quantitation of pro-COL1A1 secretion by ELISA in HFF-1 cells under the same conditions (*n* = 3). (I) Microscopy was used to observ the morphology of macrophages undergoing polarization to the M1 phenotype after treatment with 313EVs. Scale bar, 20 μm. (J) ELISA-based measurement of IL-6 secretion by ELISA in RAW 264.7 cells after M1 polarization and 313EV treatment (*n* = 3, **P < 0.01). (K) ELISA-based measurement of TNF-α secretion under the same conditions (*n* = 3). *P < 0.05, **P < 0.01, ***P < 0.001.

To comprehensively evaluate the therapeutic potential of 313EVs, the effects of 313EVs on cellular DNA damage, cellular senescence, and wound healing were investigated. After UVB irradiation, the HFF-1 cells were stained according to the instructions of the γ-H2AX kit. The degree of DNA damage in cells increased significantly after UVB irradiation. Pretreatment with 313EVs effectively reduced the degree of DNA damage compared to the UVB-irradiated group and the 205EV pretreatment group (Fig. 3A and B). In addition, senescence-associated β-galactosidase (SA-β-gal) staining was performed on HFF-1 cells after UVB irradiation. The results showed that UVB irradiation significantly increased cellular senescence. In contrast, the degree of cellular senescence in the 313EV-treatment group was significantly reduced compared to the UVB-irradiated group and the 205EV pretreatment group (Fig. 3C and D). Furthermore, 313EV treatment significantly restored the migratory capacity of HaCaT cells compared to the UVB-irradiated group, indicating that 313EV treatment can significantly repair UVB-induced skin damage (Fig. 3E and F). UVB irradiation significantly suppressed collagen type I α 1 (Col1a1) transcription in HFF-1 fibroblasts, reducing mRNA expression to 53.96% of baseline at 24 h and to 24.74% at 48 h post-exposure. In contrast, 313EV pretreatment (1E10 particles/ml) markedly rescued *COL1A1* expression, with 21.4% and 31.0% increases at 24 and 48 h, respectively, compared to UV-damaged controls (Fig. 3G). Procollagen type I α 1 (pro-Col1a1) secretion, assessed by ELISA, was similarly reduced by UVB exposure to 25.63% and 28.33% of baseline at 24 and 48 h, respectively. Pretreatment with 313EVs significantly restored pro-Col1a1 levels to 61% and 80% of unirradiated controls at the corresponding time points, indicating effective preservation of collagen biosynthesis and ECM integrity (Fig. 3H). To investigate the anti-inflammatory capacity of 313EVs, RAW cells were treated with LPS for 24 h to polarize them into the M1 phenotype and then treated with 313EVs for another 24 h. RAW cells treated with 313EV exhibited a typical M2 phenotype characterized by a loose arrangement of mainly spindle-shaped cells. In contrast, cells treated with 205EVs appeared plump with clear edges, predominantly representing the M1 phenotype. This suggests that 313EV treatment is capable of inducing a phenotypic transition of M1 macrophages toward the M2 phenotype. (Fig. 3I). Furthermore, M1 polarization led to marked increases in proinflammatory cytokine secretion, with IL-6 and TNF-α levels elevated to 6.62-fold and 13.17-fold, respectively, compared to nonpolarized controls. Treatment with 313EVs substantially attenuated this inflammatory response. At 1E10 particles/ml, IL-6 and TNF-α secretion decreased by 15.5% and 12.8% of M1 control levels, respectively. (Fig. 3J and K).

### 313EVs attenuate UV-induced dermal wrinkles

**Fig. 4.**
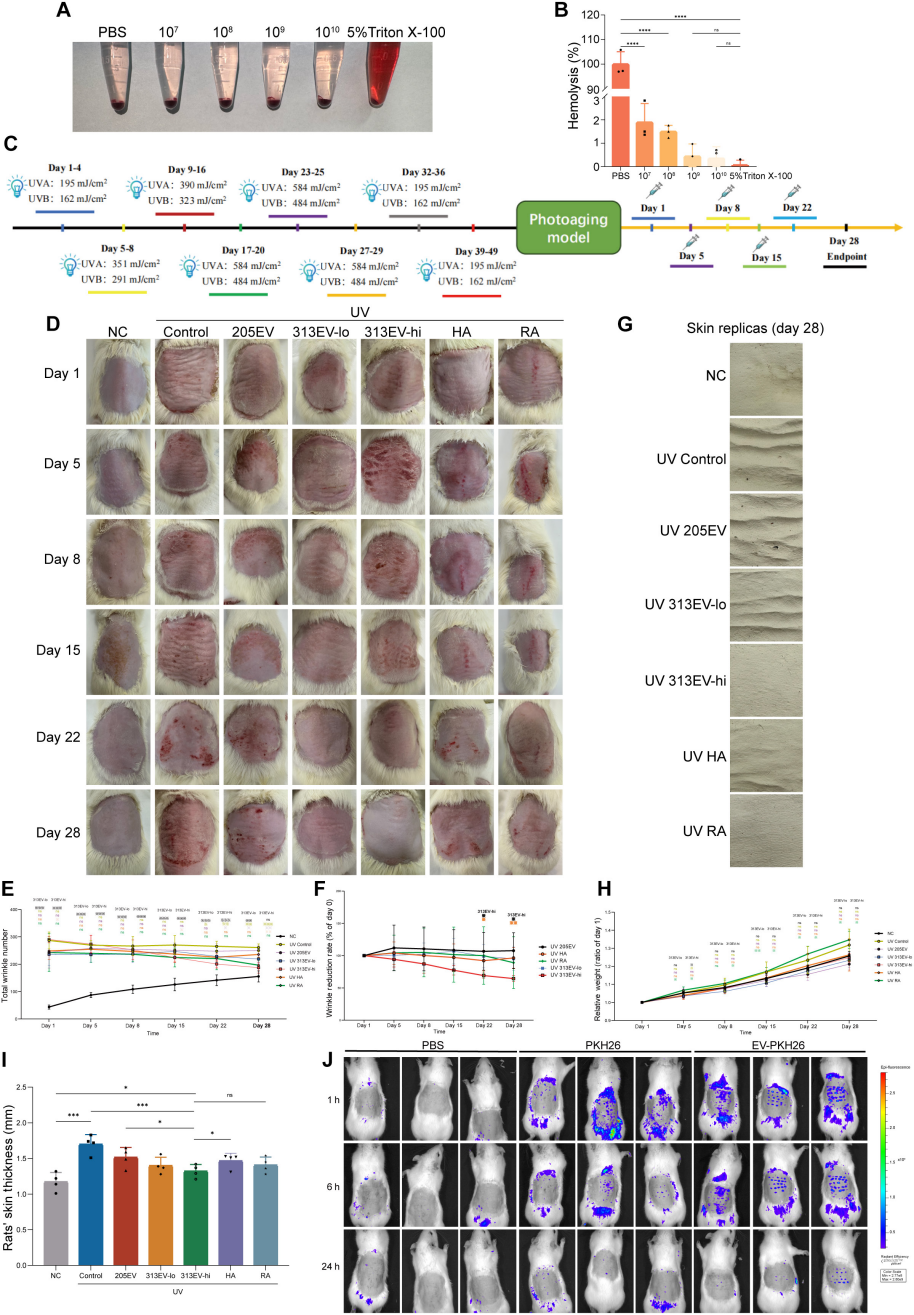
313EVs mitigate photoaging in vivo. (A and B) Hemolysis assay was employed to evaluate the cytotoxicity of the EVs. (C) Schematic illustration of the photoaging model establishment and treatment regimen. (D) Representative photographs of the dorsal skin of SD rats captured on days 1, 5, 8, 15, 22, and 28. Skin replicas were collected on day 28 (*n* = 4). (E and F) Quantification analysis of wrinkle number on the dorsal skin of rats during the treatment period (*n* = 4). (G) Representative images of dorsal skin replicas obtained on day 28. (H) Body weight measurements of rats across treatment groups throughout the study (*n* = 4). (I) Skin thickness measurements from dorsal skin sections harvested on day 28 post-treatment (*n* = 4). (J) In vivo fluorescence imaging of rats following intradermal injection of PKH26-labeled 313EVs, showing EV retention in the dermis at 24 h (*n* = 4). *P < 0.05, **P < 0.01, ***P < 0.001.

To evaluate the therapeutic efficacy of 313EVs for treating UV-induced photoaging in vivo, we first investigated its biosafety profile. Hemolysis assays were conducted to evaluate the cytotoxicity of different concentrations of 313EVs. In the 5% Triton X-100 group, hemolysis was evident, with the supernatant appearing red after centrifugation and a significant number of red blood cells lysed. The 313EV-treated group and the PBS control showed no signs of hemolysis, with clear supernatants after centrifugation. The optical density (OD) results indicated that none of the tested concentrations of 313EVs induced hemolysis, with hemolysis rates remaining below 2%, thereby demonstrating the favorable biocompatibility of 313EVs (Fig. 4A and B). Following successful model establishment, treatments were administered on days 1, 5, 8, 15, 22, and 28. Rats were photographed and weighed at each time point (Fig. 4C). At the end of the study, dorsal skin samples were collected for histological analysis and skin replica casting (Fig. 4D). Beginning on day 22, the high-dose 313EVs group exhibited a significantly greater reduction in wrinkle formation compared to the 205EVs and HA groups, with further improvement noted by day 28. These findings indicate that intradermal administration of 313EVs effectively improves skin condition and reduces photoaging-induced wrinkles (Fig. 4E and F). Skin replicas obtained on day 28 confirmed the superior anti-wrinkle efficacy of 313EVs relative to the UV control, 205EVs, HA, and retinoic acid (RA) control groups (Fig. 4G). Body weight remained stable across all groups throughout the treatment period, suggesting good tolerability of 313EVs (Fig. 4H). Remarkably, high-dose 313EV treatment over 28 d markedly reduced dermal thickening, with therapeutic outcomes surpassing those of the RA group (Fig. 4I), underscoring the potent anti-photoaging effect of 313EVs. To assess the local distribution of EVs, PKH26-labeled 313EVs were intradermally injected into rats, followed by in vivo fluorescence imaging. Fluorescent signals remained detectable in the dermis at 24 h post-injection, confirming effective tissue retention of 313EVs (Fig. 4J).

### Effects of 313EVs on pathological features and collagen content in vivo

**Fig. 5.**
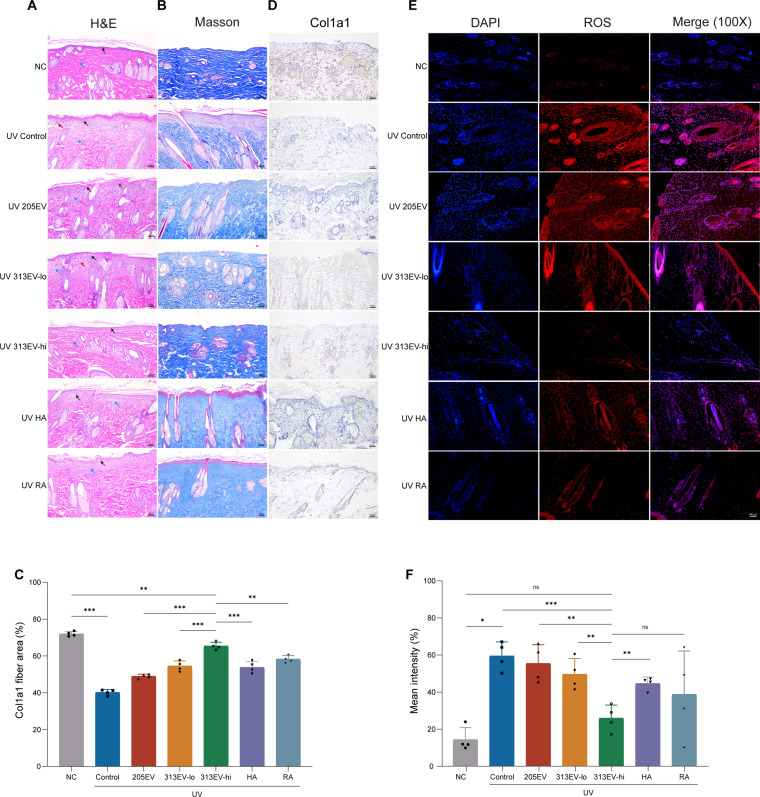
313EVs mitigate photoaging in vivo. (A and B) Representative images of H&E staining and Masson’s trichrome staining of dorsal skin sections from each treatment group. Scale bar, 100 μm. (C) Quantification analysis of collagen fiber area reveals a significant increase in collagen deposition in the 313EV-treated group. (D) Representative images of Col1a1 immunohistochemical staining of skin sections from the back of each treatment group. Scale bar, 100 μm. (E) Representative images of DHE staining for detection of ROS. Scale bar, 100 μm. (F) Quantification of ROS content based on red fluorescence intensity following DHE staining.

To evaluate the histopathological impact of 313EV treatment on photoaged skin, H&E staining was performed on dorsal skin samples from SD rats. In the UV-irradiated control group, marked epidermal erosion, necrosis, and extensive inflammatory cell infiltration were observed, consistent with characteristic features of photoaging and photodamage. In contrast, 313EV treatment significantly attenuated these pathological changes, reducing epidermal injury and inflammation while restoring dermal collagen fiber structure and density (Fig. 5A). To assess collagen deposition, skin samples collected on day 28 post-treatment were subjected to Masson’s trichrome staining, followed by quantitative analysis for collagen content. Compared to HA and RA treatments, 313EVs administration led to a pronounced increase in collagen synthesis and remodeling, reflecting its superior capacity to promote tissue repair and matrix regeneration (Fig. 5B and C). Immunohistochemical analysis was performed to assess the collagen content in skin sections. The 313EV-treatment group exhibited significantly improved regeneration of Col1a1 compared to the untreated group, with better skin and collagen structure than those in the HA and RA treatment groups (Fig. 5D). Additionally, the antioxidant properties of 313EVs were investigated by DHE staining to quantify ROS levels in skin tissues. Fluorescence imaging and quantitative analysis revealed a significant reduction in ROS accumulation in the 313EV-treated groups, with the high-dose group (313EV-hi) demonstrating more potent ROS scavenging activity than the low-dose group (313EV-lo), indicating a dose-dependent antioxidant effect (Fig. 5E and F).

### Effects of 313EVs on gene expression and protein secretion in vivo

**Fig. 6.**
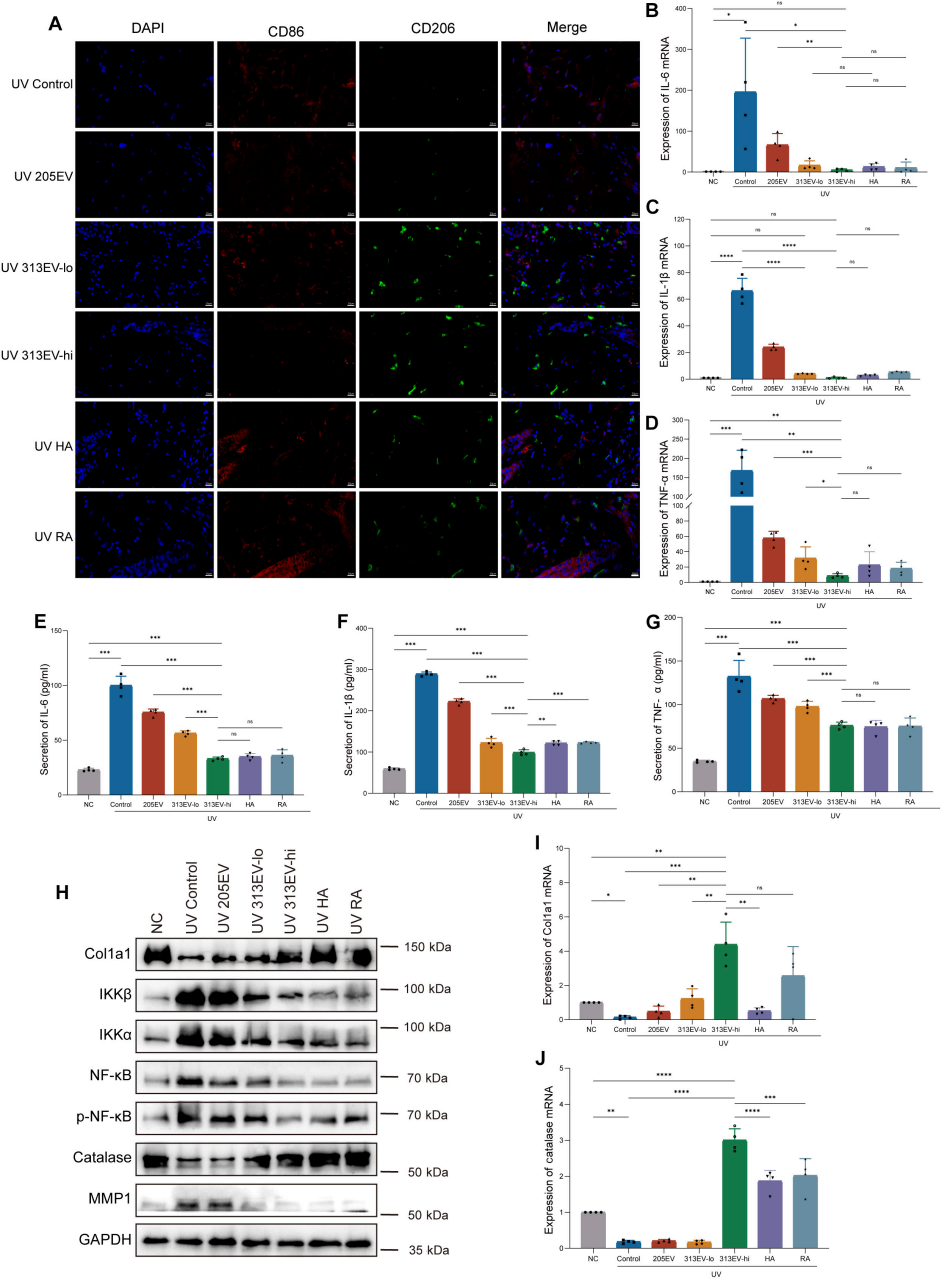
313EVs alleviate UV-induced skin photoaging in vivo. (A) Representative images of immunofluorescence for CD206 and CD86 in skin sections from the back of each treatment group. Scale bar, 20 μm. (B to D) Quantitation RT-qPCR analysis of IL-6, IL-1β, and TNF-α mRNA expression in rat dorsal skin following 313EV treatment (*n* = 4). (E to G) ELISA quantitation of IL-6, IL-1β, and TNF-α protein levels in serum and skin tissue (*n* = 4). (H) Western blots analysis of NF-κB signaling-related proteins (NF-κB, IKKα, IKKβ, and p-NF-κB), Col1a1, CAT, and MPP1 in skin tissue across treatment groups. (I and J) RT-qPCR quantitation of Col1a1 and CAT mRNA expression (*n* = 4). *P < 0.05, **P < 0.01, ***P < 0.001.

To investigate the anti-inflammatory effects of 313EVs, immunofluorescence was used to examine the levels of CD206 and CD86 in skin sections. In the 313EV-treated group, the immunofluorescence sections showed a substantial expression of green fluorescence for CD206, while the expression of CD86 was significantly reduced compared to the untreated group. The green fluorescence of CD206 in the 313EV-treatment group was significantly increased compared to other treatment groups, while CD86 showed the opposite trend. This indicates that 313EV treatment has the effect of transforming proinflammatory M1 macrophages into anti-inflammatory M2 macrophages. The results indicated that the low-dose 313EV-treatment group also exhibited significant therapeutic effects. The 313EV-treatment group demonstrated superior therapeutic effects in reversing macrophage phenotypes compared to the commonly used drugs HA and RA (Fig. 6A). In addition, to investigate the therapeutic efficacy of 313EVs in vivo, we systematically analyzed their impact on collagen remodeling and anti-inflammatory responses in a UV-induced photoaging rat model. RT-qPCR analysis demonstrated that 313EV treatment significantly down-regulated mRNA expression of proinflammatory cytokines IL-1β, IL-6, and TNF-α compared to the control group. Notably, even low-dose 313EV treatment led to substantial reductions in inflammatory gene expression. In the high-dose group, inflammatory cytokines (IL-6, IL-1β, and TNF-α) mRNA levels were comparable to those in the NC group, suggesting effective suppression of UV-induced inflammation (Fig. 6B to D). Consistent with the mRNA findings, ELISA results showed a significant dose-dependent reduction in IL-1β, IL-6, and TNF-α protein levels in both serum and skin tissues of 313EV-treated rats (Fig. 6E to G). These results indicate that 313EVs exert robust anti-inflammatory effects, attenuating the elevated cytokine levels associated with UV-induced photoaging.

To explore the underlying mechanisms, Western blot analysis was performed to assess the activation state of the nuclear factor-κB (NF-κB) signaling pathway. Compared to the control group, 313EV treatment resulted in decreased expression of NF-κB, IκB kinase α (IKKα), IKKβ, and phosphorylated NF-κB (p-NF-κB), suggesting that 313EVs suppress UV-induced NF-κB activation (Fig. 6H). Given that NF-κB signaling drives matrix metalloproteinase-1 (MMP-1) expression and collagen degradation, we further examined collagen homeostasis. Western blot results demonstrated down-regulation of MMP-1 and concurrent up-regulation of collagen I protein in 313EV-treated skin. Moreover, RT-qPCR analysis revealed a 4.38-fold increase in Col1a1 mRNA expression in the high-dose 313EV-treated group compared to controls, confirming enhanced collagen synthesis (Fig. 6I). To assess the antioxidant capacity of 313EVs, we measured CAT expression levels. Both CAT mRNA and protein levels were significantly elevated in the 313EV-treated group compared to controls, indicating enhanced ROS-scavenging capacity (Fig. 6J). Collectively, these findings suggest that 313EVs alleviate photoaging through a multifaceted mechanism involving NF-κB pathway inhibition, reduction of proinflammatory cytokines, restoration of collagen synthesis, and reinforcement of the skin’s antioxidant defense system.

### In vivo proteomic insights into the therapeutic effects on photoaged skin

**Fig. 7.**
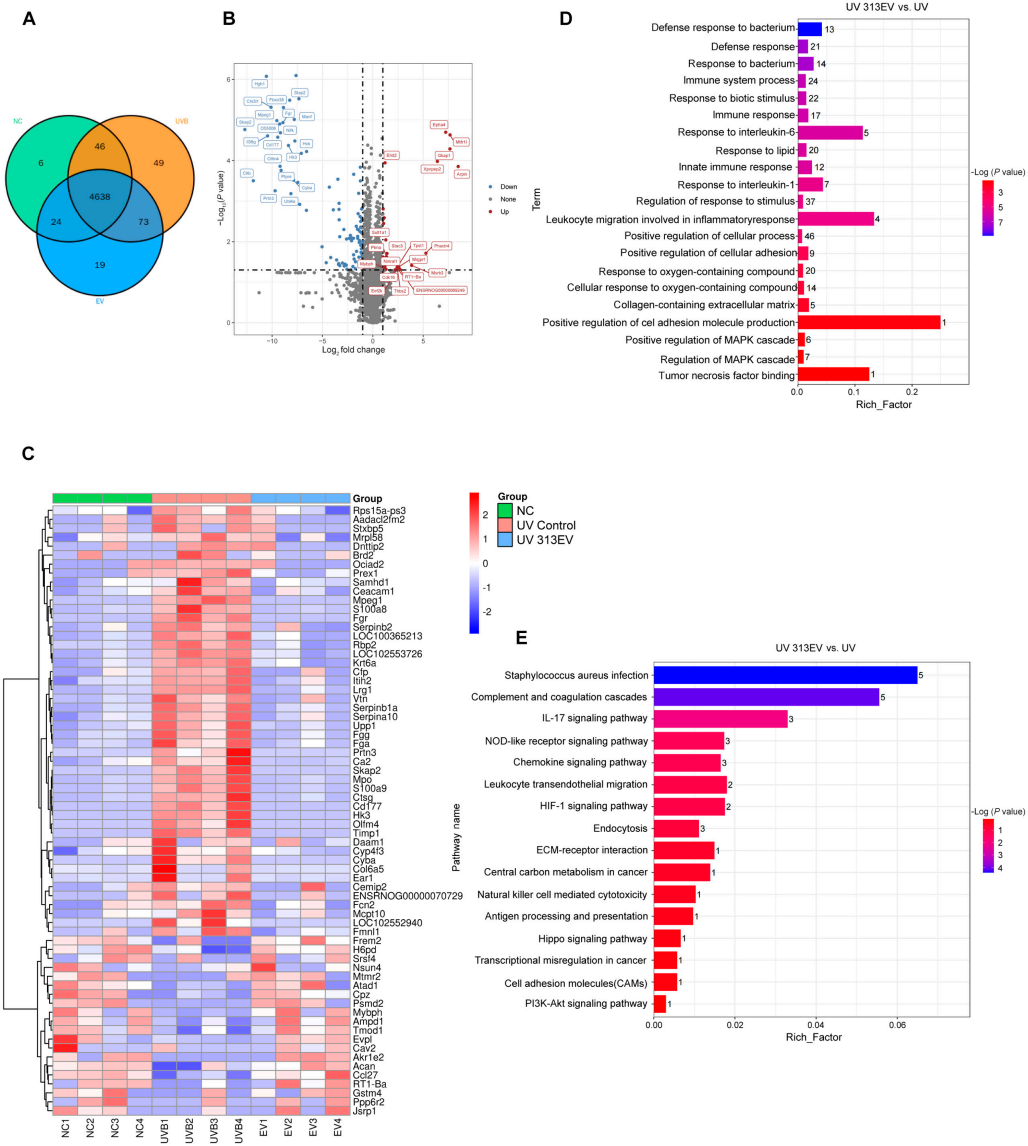
Proteomic analysis of molecular responses to 313EV therapy in photoaged skin. (A) Venn diagram illustrating the overlap and unique protein expression profiles among the normal control (NC), UVB-induced photoaged, and 313EV-treated groups. (B) Volcano plot displaying differentially expressed proteins across NC, UVB, and 313EV-treatment groups, highlighting significant up-regulation and down-regulation patterns. (C) Heatmap of hierarchical clustering showing protein expression profiles and group-specific changes in response to UVB irradiation and 313EV treatment. (D) GO enrichment analysis revealing significant biological processes modulated by 313EV treatment, including MAPK cascade regulation and inflammatory cytokine responses. (E) KEGG pathway enrichment analysis identifying key signaling pathways involved in the repair of photoaged skin, such as PI3K–Akt, HIF-1, and cell adhesion molecule pathways, following 313EV therapy.

To elucidate the molecular mechanisms underlying photoaging and the therapeutic effects of 313EVs, we conducted in vivo proteomic profiling of skin tissues collected from normal control, UVB-induced photoaged, and 313EV-treated rats. Venn diagram analysis identified 24 proteins shared between the normal control and EV-treated groups, which were absent in the UVB group—suggesting that 313EVs may restore protein expression patterns altered by photoaging. Additionally, 19 proteins were uniquely expressed in the EV-treated group, indicating treatment-specific molecular responses (Fig. 7A). The distribution of differentially expressed proteins was depicted in Fig. 7B.

Notably, 313EV treatment led to significant up-regulation of several proteins associated with oxidative stress response and tissue repair. Prothymosin α (Ptma), a protein involved in antioxidant defense and DNA repair, was markedly elevated, suggesting the activation of protective cellular pathways. Methionine sulfoxide reductase B3 (Msrb3), an enzyme that reverses oxidative protein modifications, was also significantly increased, indicating enhanced ROS detoxification. Moreover, eukaryotic elongation factor 2 kinase (Eef2k), a stress-responsive kinase that regulates translation under oxidative conditions, exhibited elevated expression, highlighting a shift toward cellular survival and energy conservation. Up-regulation of SH3 and cysteine-rich domain-containing protein 3 (Stac3), which modulates calcium signaling, further supports the involvement of adaptive stress responses essential for tissue homeostasis.

Gene expression analysis also revealed distinct molecular signatures associated with photoaging and its resolution. In the UVB group, genes such as Cd177, S100a9, Olfm4, and Prtn3, which are closely linked to inflammatory responses and ECM degradation, were significantly up-regulated, reflecting the pathological impact of photoaging. In contrast, 313EV treatment promoted the expression of proteins like Mpeg1, Fgr, Akr1e2, and Cav2, which are involved in antioxidant defense, membrane signaling, and tissue regeneration. These findings suggest that 313EVs mitigate photoaging by suppressing proinflammatory gene expression while enhancing pathways related to cellular protection and repair (Fig. 7C).

GO enrichment analysis further revealed that 313EV treatment activated biological processes related to mitogen-activated protein kinase (MAPK) cascade regulation, inflammatory cytokine responses (including TNF, IL-1, and IL-6 signaling), immune cell activation, and ECM organization. These results imply that 313EVs promote dermal regeneration by reducing inflammation, mitigating oxidative stress, enhancing immune cell function, and supporting collagen-rich matrix remodeling (Fig. 7D). KEGG pathway analysis confirmed the activation of key signaling cascades relevant to skin repair. Notably, phosphatidylinositol 3-kinase (PI3K)–Akt signaling, central to collagen synthesis and ECM remodeling, was significantly enriched. Additionally, enhanced activity in the cell adhesion molecule (CAM) and HIF-1 signaling pathways supports improved immune cell recruitment and angiogenesis, both of which are critical for epidermal repair (Fig. 7E).

Collectively, these proteomic and transcriptomic analyses demonstrate that 313EVs exert potent anti-photoaging effects by reprogramming the molecular landscape of damaged skin, activating protective signaling networks, and restoring tissue integrity.

### Effects of skin microbial flora on 313EV-mediated photoaging repair in vivo

**Fig. 8.**
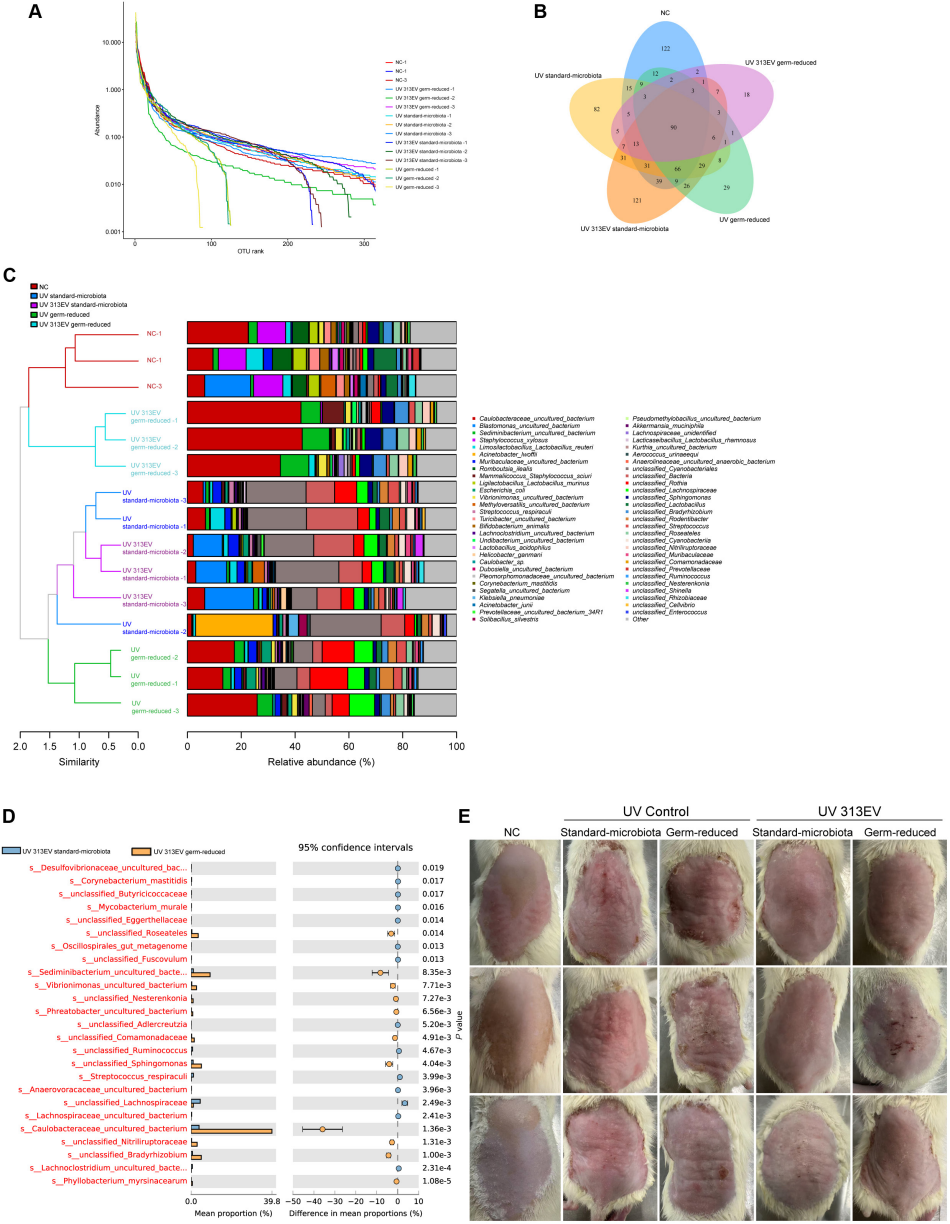
The role of skin microbiota in 313EV-mediated photoaging therapy. (A) Rank–abundance curves showing species richness and distribution across samples. (B) Venn diagram illustrating the overlap or uniqueness of microbial species among groups. (C) Combined hierarchical clustering and bar-chart analysis demonstrating intergroup differences in microbial composition. (D) Differential abundance analysis of microbial taxa between groups. The central bar indicates the 95 % confidence interval of the abundance ratio, and the value on the right represents the corresponding *P* value (*P* < 0.05 denotes statistical significance). Red highlights indicate the top 25 differentially enriched taxa. (E) Representative dorsal skin images of SD rats from each treatment group.

To reduce potential microbial interference with 313EV-mediated therapeutic effects, we established a rat model with markedly diminished skin microbiota. 16*S* rRNA sequencing of skin swab samples confirmed a clear reduction in microbial abundance and species richness in the germ-reduced group compared with the standard-microbiota group (Fig. 8A and B). Differential abundance analysis further identified microbial taxa that varied significantly between the 313EV germ-reduced and the 313EV standard-microbiota groups (Fig. 8C). However, none of these taxa were associated with known roles in skin repair, inflammation, or UV-induced tissue damage, suggesting limited biological relevance to the photoaging process (Fig. 8D).

We next evaluated whether microbiota depletion influenced the therapeutic efficacy of 313EVs. Comparative imaging of dorsal skin morphology revealed no notable differences in the extent of wrinkle reduction, erythema attenuation, or tissue restoration between germ-reduced animals and those with intact skin microbiota following 313EV administration. Thus, the anti-photoaging effects of 313EVs were comparable in both groups (Fig. 8E).

Taken together, these findings indicate that, under the conditions of our study, endogenous skin microbiota exert minimal impact on 313EV-mediated photodamage repair. The therapeutic actions of 313EVs therefore appear to be driven predominantly by vesicle-intrinsic mechanisms rather than by microbe–host interactions.

## Discussion

EVs have emerged as next-generation delivery systems owing to their intrinsic biocompatibility, ability to traverse biological barriers, and reduced clearance by the mononuclear phagocyte system compared with synthetic nanocarriers [29–31]. While EV-based encapsulation of small-molecular antioxidants such as vitamin C and resveratrol has been reported, the efficient delivery of large enzymatic proteins (e.g., CAT, ∼240-kDa tetramer) remains highly challenging because of their structural complexity and propensity for denaturation during loading [32,33]. To overcome these limitations, we developed a bioengineered exosome platform, termed 313EVs, for the targeted delivery of CAT to photoaged skin. We first optimized a HEK293-derived producer line (205 cells) with high EV secretion capacity. Through lentiviral transduction of a CAT-expressing construct followed by stringent antibiotic selection, we established a stable subclone designated 313 cells, which maintained the vesicle production capacity of parental 205 cells while exhibiting robust intracellular CAT overexpression. Mass spectrometry analysis confirmed substantial CAT enrichment in EVs secreted by 313 cells, demonstrating the feasibility of intracellular cargo loading via genetic engineering rather than post-isolation modification, which often compromises protein bioactivity.

Across in vitro and in vivo models of photoaging, 313EVs demonstrated pronounced therapeutic efficacy. In vivo, intradermal administration of 313EVs visibly improved the macroscopic appearance of UVB-damaged rat skin, attenuated wrinkle formation, reduced erythema, and elicited no adverse effects on body weight, indicating favorable biosafety. Histological assessments further showed that 313EVs restored dermal collagen architecture, suppressed inflammatory infiltration, and significantly decreased tissue ROS levels. The reduction of γ-H2AX foci and SA-β-gal staining suggested that 313EVs alleviate both DNA damage and cellular senescence. In vitro wound-healing assays additionally demonstrated that 313EVs enhanced the migratory capacity of UVB-exposed HaCaT cells, supporting their role in promoting post-photodamage tissue repair. Mechanistically, 313EV treatment reduced secretion of the proinflammatory cytokines IL-1β, IL-6, and TNF-α by down-regulating the NF-κB pathway activation while simultaneously promoting collagen synthesis and regenerative processes.

HEK293-derived EVs were efficiently internalized by cutaneous epithelial cells, which enhances their translational applicability for human skin therapy. Importantly, because 313EVs are produced directly by genetically modified cells rather than being manipulated post-isolation, their biocompatibility is superior to that of EVs subjected to exogenous loading procedures. GO and KEGG analyses further confirmed the absence of teratogenic or tumorigenic signatures and revealed enrichment in cell adhesion-related proteins, suggesting efficient uptake via ECM receptor-mediated interactions and focal adhesion pathways.

The central rationale for engineering CAT-enriched 313EVs was to counteract UV-induced oxidative stress, a major driver of photoaging. In vitro, 313EVs suppressed intracellular H_2_O_2_ accumulation and improved viability in both UVB-irradiated and H_2_O_2_-challenged cells, outperforming commercially available mesenchymal stem cell-derived EVs (huMSC-EVs). In addition to ROS neutralization, 313EVs promoted collagen synthesis and substantially reduced inflammatory cytokine release, underscoring their multifaceted therapeutic capacity.

Our use of both prophylactic (in vitro) and therapeutic (in vivo) treatment paradigms was intentional, reflecting the distinct temporal dynamics of oxidative damage. Acute UVB injury generates immediate bursts of hydroxyl radicals (·OH), requiring rapid antioxidant intervention [34,35]. In contrast, chronic photoaging involves long-term ROS-driven alterations, including protein carbonylation and mitochondrial DNA lesions, that necessitate sustained reparative therapies [36–38]. The success of 313EVs in both contexts underscored the versatility of EV-based antioxidant delivery systems.

Proteomic analyses provided further mechanistic insight, revealing that 313EV treatment up-regulated proteins involved in antioxidant defense (e.g., Ptma and Msrb3), translational regulation (Eef2k), and calcium signaling (Stac3) while down-regulating inflammation- and ECM-degrading proteins (e.g., S100a9 and Prtn3). Enrichment analyses implicated pathways associated with MAPK regulation, PI3K–Akt signaling, and ECM organization—all consistent with a repair-focused molecular response.

As research into photoaging increasingly highlights the regulatory importance of miRNAs, our RNA-sequencing analyses offer additional mechanistic clarity. The miRNA cargo of 313EVs was enriched in pathways related to cellular repair, metabolic recovery, and chromatin regulation, suggesting that these miRNAs may synergistically support CAT-mediated antioxidant effects. Notably, genetic modification of 205 cells to generate 313 cells subtly reshaped the miRNA repertoire of their secreted EVs, indicating an additional layer of therapeutic modulation.

In contrast, the contribution of the skin microbiota to 313EV-mediated repair appeared minimal. Although the skin microbiome is known to influence epidermal homeostasis, immune responses, and barrier function, our germ-reduced animal model demonstrated no significant difference in anti-photoaging efficacy between microbiota-depleted and microbiota-intact conditions. Moreover, the bacterial taxa that were differentially abundant between groups lacked established roles in skin repair or photoaging. These findings indicate that, under our experimental conditions, the skin microbiome did not meaningfully modulate the therapeutic effects of 313EVs.

In summary, we developed a genetically engineered CAT-enriched EV platform and demonstrated its potent antioxidant, anti-inflammatory, and matrix-restorative activities in models of photoaging. The combination of enhanced ROS scavenging, ECM preservation, and improved cellular repair positions 313EVs as a promising, biocompatible candidate for clinical translation in the treatment and prevention of photoaging.

## Data Availability

The datasets used and/or analyzed during the current study are available from the corresponding author upon reasonable request.
